# Evaluation of the toxicity and repellence of an organic fatty acids mixture (C8910) against insecticide susceptible and resistant strains of the major malaria vector *Anopheles funestus* Giles (Diptera: Culicidae)

**DOI:** 10.1186/s13071-015-0930-2

**Published:** 2015-06-12

**Authors:** Michael Samuel, Shüné V. Oliver, Oliver R. Wood, Maureen Coetzee, Basil D. Brooke

**Affiliations:** Wits Research Institute for Malaria, School of Pathology, Faculty of Health Sciences, University of the Witwatersrand, Johannesburg, South Africa; Centre for Opportunistic, Tropical & Hospital Infections, National Institute for Communicable Diseases, Johannesburg, South Africa

**Keywords:** *Anopheles funestus*, C8910 toxicity, C8910 repellence, malaria vector control

## Abstract

**Background:**

Malaria vector control relies principally on the use of insecticides, especially pyrethroids. Because of the increasing occurrence of insecticide resistance in target vector populations, the development of new insecticides, particularly those with novel modes of action, is particularly important, especially in terms of managing insecticide resistance. The C8910 formulation is a patented mixture of compounds comprising straight-chain octanoic, nonanoic and decanoic saturated fatty acids. This compound has demonstrated toxic and repellent effects against several arthropod species. The aims of this study were to measure the insecticidal effects of C8910 against an insecticide susceptible (FANG) and a pyrethroid resistant (FUMOZ-R) laboratory strain of *An. funestus* as well as against wild-caught *An. funestus* material from Zambia (ZamF), and to investigate the repellent effects of two formulations of C8910 against these strains.

**Methods:**

Toxicity against adult females was assessed using a range of concentrations based on the CDC bottle bioassay method and repellence of three different C8910 formulations was assessed using standard choice-chamber bioassays.

**Results:**

C8910 proved equally toxic to adult females of the FUMOZ-R and FANG laboratory strains, as well as to adult females of the wild-caught (ZamF) sample. None of the C8910 formulations tested gave any conclusive indication of repellence against any of the strains.

**Conclusion:**

C8910 is equally effective as an adulticide against pyrethroid resistant and insecticide susceptible *An. funestus*. However, the formulations tested did not show any consistent repellence against laboratory reared and wild-caught female samples of this species. Nevertheless, C8910 shows potential as an adulticide that can be used for malaria vector control, particularly in those instances where insecticide resistance management is required.

## Background

The World Health Organization (WHO) estimates that 198 million cases of malaria and 584,000 resultant deaths occurred globally in 2013 [1]. The vast majority of malaria cases occur in the Afrotropical region.

*Anopheles funestus* Giles is a primary malaria vector species in the Afrotropical region [2, 3] and is the nominal member of the Funestus Subgroup which comprises four species: *An. funestus, An. parensis, An. vaneedeni* and *An. confusus*. These are almost morphologically indistinguishable at all life stages [3, 4]. Of these, only *An. funestus* has been implicated in malaria transmission. *Anopheles funestus* females are highly anthropophilic and endophilic, and often take multiple blood meals. These characteristics combined with a relatively high longevity make *An. funestus* populations especially efficient at malaria transmission, and often show higher *Plasmodium falciparum* sporozoite infection rates than other vector species [5].

A strong tendency toward endophily makes *An. funestus* populations especially susceptible to control by indoor spraying of residual insecticides (IRS). Currently, only insecticides belonging to the pyrethroid, carbamate, organophosphate and organochlorine (DDT only) classes are available for malaria vector control. These collectively target only two insect neurological sites (the sodium ion channel and acetylcholinesterase) [6], which makes the development of resistance and cross-resistance between classes a likely prospect in regions where insecticide selection is suitably intense. The rate of resistance development is also exacerbated by the use of these insecticides for agricultural pest control [7]. Several populations of *An. funestus* have developed resistance to insecticides including pyrethroids (type I and II), carbamates (bendiocarb and propoxur), the organochlorine DDT and the cyclodiene dieldrin (reviewed by Coetzee & Koekemoer [8]).

The many instances of insecticide resistance in *An. funestus* highlight a significant problem facing insecticide based vector control strategies, the consequences of which are exemplified by the malaria epidemic of 1996–2000 experienced in northern KwaZulu-Natal, South Africa [9, 10]. During this epidemic, pyrethroid and carbamate resistant *An. funestus* were able to expand their ranges into areas that were under pyrethroid-based IRS control [11]. The epidemic was subsequently halted by a range of interventions including the re-introduction of DDT into the IRS programme as a resistance management option [10]. Insecticide resistance in malaria vector populations has become so widespread that malaria vector control is synonymous with resistance management, and has led to the development of the Global Plan for Insecticide Resistance Management (GPIRM) [12].

Since the introduction of pyrethroids in the 1990s, no new classes of insecticide have been approved by the WHO for use in public health [13]. The development of new insecticides, particularly those with novel modes of action, is particularly important, especially in terms of managing insecticide resistance [14].

In combination with insecticide applications, topical and spatial insect repellents are potentially useful in malaria vector control [15, 16]. Several candidate repellents have recently been identified [16]. However, their use as alternatives is not widespread, and a reduction in the efficacy of repellents can occur over time [17].

Fatty acids found naturally on human skin have shown repellent effects against *Aedes aegypti* mosquitoes including those attempting to bite or oviposit [18–20]. The C8910 formulation is a patented mixture of compounds comprising straight-chain octanoic, nonanoic and decanoic saturated fatty acids (C8, C9 and C10) [21]. This formulation has demonstrated repellent effects against biting and non-biting flies as well as ticks [22]. In addition, an incapacitating and toxic effect against several mosquito species, including the dengue vector *Ae. aegypti* [20] and several malaria vector species including *An. gambiae, An. dirus, An. farauti, An. freeborni, An. minimus* and *An. stephensi* [23], has been observed. The mode of action of C8910 has not been fully elucidated but likely involves respiratory inhibition [21].

The aims of this study were to measure the insecticidal effects of C8910 against insecticide susceptible and pyrethroid resistant laboratory strains of *An. funestus* as well as against wild-caught *An. funestus* from Zambia, and to investigate the repellent effects of two formulations of C8910 against these strains.

## Methods

### Mosquito strains

All of the mosquito strains/samples used in this study are housed in the Botha De Meillon Insectary (BMDI) at the National Institute for Communicable Diseases (NICD) in Johannesburg.

Ethical clearance for the use of mosquitoes for research purposes has been obtained from the Human Research Ethics Committee (medical) of the University of the Witwatersrand, Johannesburg (ref: W-CJ-100510-1).

#### Laboratory-reared colonies

FANG: This *An. funestus* colony originated from southern Angola and has been kept in colony since 2003. It is fully susceptible to insecticides. Specifically, adult males and females exposed to 0.75 % permethrin for 1 h consistently show 100 % mortality 24 h post exposure.

FUMOZ-R: This *An. funestus* colony has been selected for pyrethroid resistance from the base colony (FUMOZ) which originated from southern Mozambique and has been kept in colony since July 2001 [24].

#### Wild-caught samples

ZamF: Samples of indoor-resting *An. funestus* Clade I, highly resistant to pyrethroids and carbamates yet susceptible to DDT and organophosphates, were collected in the Nchelenge District of Zambia during the first quarter of 2014 [25]. Females were transported live to the BDMI where they were induced to lay eggs. Hatched larvae were reared through to adulthood and samples of F1 adult progeny (as representative of the wild population) were used for the toxicity and repellence tests detailed below.

### Toxicity assays

The CDC bottle bioassay protocol of Brogdon & Chan [26] was used to assess the insecticidal effects of C8910 on the *An. funestus* colonies/wild-caught samples. C8910 was supplied in liquid form by the Centers for Disease Control and Prevention (CDC; Atlanta, Georgia) and diluted in acetone to obtain a series of dilutions at a concentration range of 50-400 μg a.i./ml. 1 ml of each C8910 solution was used to treat each bottle and controls included bottles treated with acetone only. Twenty to twenty-five adult female mosquitoes (aged 1–5 days old) were gently introduced into each bottle. Knockdown, which was defined as a mosquito on its back and unable to right itself, was recorded at 15 min intervals over a 2 h exposure period. The entire concentration range plus a control was assessed through 3 replicates per colony/wild-caught sample. Following exposure, mosquitoes were removed from the bottles and placed into holding cups with access to 10 % sucrose solution. Environmental controls were conducted concurrently with unexposed mosquitoes directly inserted into a holding cup with access to sucrose solution. Final mortalities were recorded at 24 h post-exposure.

All statistical analyses were done using IBM SPSS (Armonk, NY). The mean Lethal Concentrations inducing 50 % mortality (LC50s) were determined for each colony/wild-caught sample using regression lines of log-transformed mortality data. Analysis of variance (ANOVA) was used to compare LC50s between the three data sets. As all control deaths were below 10 %, no mortality data were corrected.

### Repellence tests

Repellence tests were conducted using standard choice-chamber bioassays. Initially, two C8910 repellent formulations developed by Stratacor Inc. (Richmond, CA) were supplied. Each of these consisted of 15 % C8910 as the active ingredient, suspended in a mineral oil carrier and scented with original green leafy (formulation 1) and modified green leafy (formulation 2) fragrances. A C8910 fatty acid blend produced by Emery Oleochemicals (Cincinnati, OH) was suspended in mineral oil to create a third formulation, also comprising 15 % C8910. This formulation was used for the repellence tests on the ZamF strain.

In order to avoid residual repellents affecting subsequent bioassays, disposable cloths were treated with 5 ml of each formulation and partially wrapped around the treatment forearm of each investigator. A cloth treated with 5 ml DC345 silicon oil was wrapped around each investigator’s alternate arm as a control. Positive control tests on each colony/wild-caught sample were conducted using a commercial product containing the insect repellent DEET (N, N-diethyl-meta-toluamide-19.5 %) as its active ingredient. The DEET product was applied to the cloth of the treatment arm of each investigator as described above. Three investigators participated in the assays for C8910 formulations 1 and 2. Owing to limited wild-caught material, only two investigators participated in the assays for C8910 formulation 3.

Forty to fifty non blood-fed adult females per strain/sample per replicate were starved of sugar solution for 12 h and then gently inserted into the choice-chamber. They were left to acclimatize for 20 min in the dark in climate-controlled conditions of 25 ± 2 °C and 75 % humidity. The investigator concerned then placed the treatment and control arms at either end of the choice-chamber, so that each arm was 2 cm from the gauze-covered end of the chamber. The number of mosquitoes landing and attempting to blood-feed (hereafter referred to as the number of landings) on either arm was recorded at 3 min intervals for a period of 15 min. During counts, a red light was used to illuminate the chamber so as to minimize disturbance. The repellence of each formulation was assessed for each colony/sample through three replicates per investigator.

Variation in the total number of landings on the control versus treatment arms at each time interval was tested for significance using paired t-tests. These analyses were done using IBM SPSS with significance set at 95 % confidence.

## Results

### C8910 toxicity

**Fig. 1:**
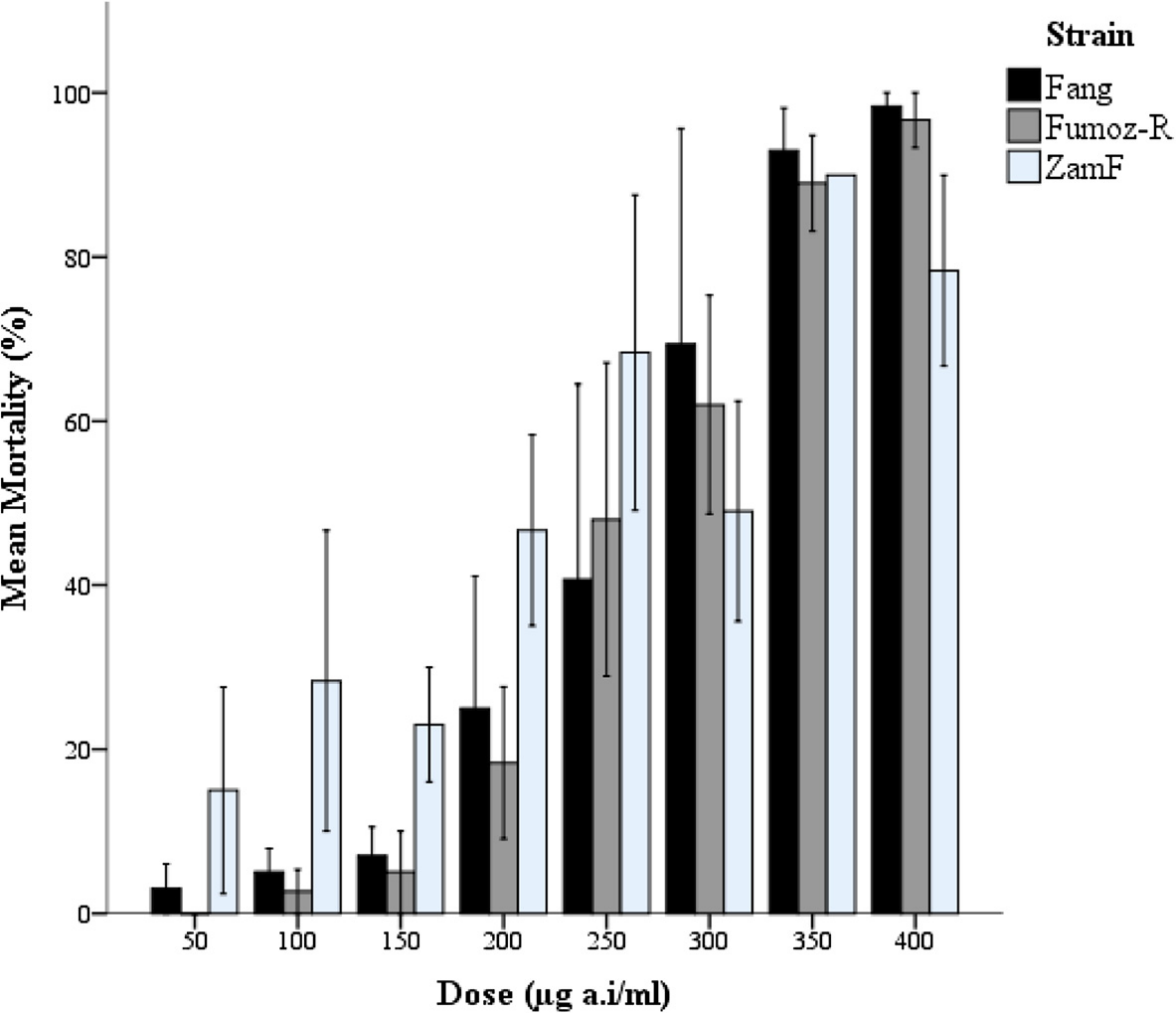
Mean percentage mortality of *Anopheles funestus* strains 24 h post-exposure to C8910. FANG (insecticide susceptible) and FUMOZ-R (pyrethroid resistant) colonies as well as ZamF (wild caught) samples were exposed to C8910-treated bottles in accordance with the CDC bottle bioassay protocol. C8910 exposure lasted 2 h, after which, the mosquitoes were removed and placed in non-treated holding containers. Mortality was recorded 24 h after the initial exposure began. Three replicates were completed per dose per strain. Only one replicate of ZamF was achieved at 350 μl/ml a.i bottle

The dose-mortality response curves for each sample are shown in Fig. 1. There was no statistically significant difference in response between the three strains (one-way ANOVA: F = 0.19, *p* = 0.83). In general, mortality increased with increasing C8910 concentration for all samples. However, this trend was least obvious in the wild-caught ZamF sample, especially at the lower doses (50-250 μg/ml a.i). A Tukey HSD post-hoc test of the ZamF 50-250 μg/ml a.i dose range shows that the significant trend (F = 3.4, *p* = 0.04) indicated by one-way ANOVA does not reflect a significant difference in mean mortalities.

**Fig. 2:**
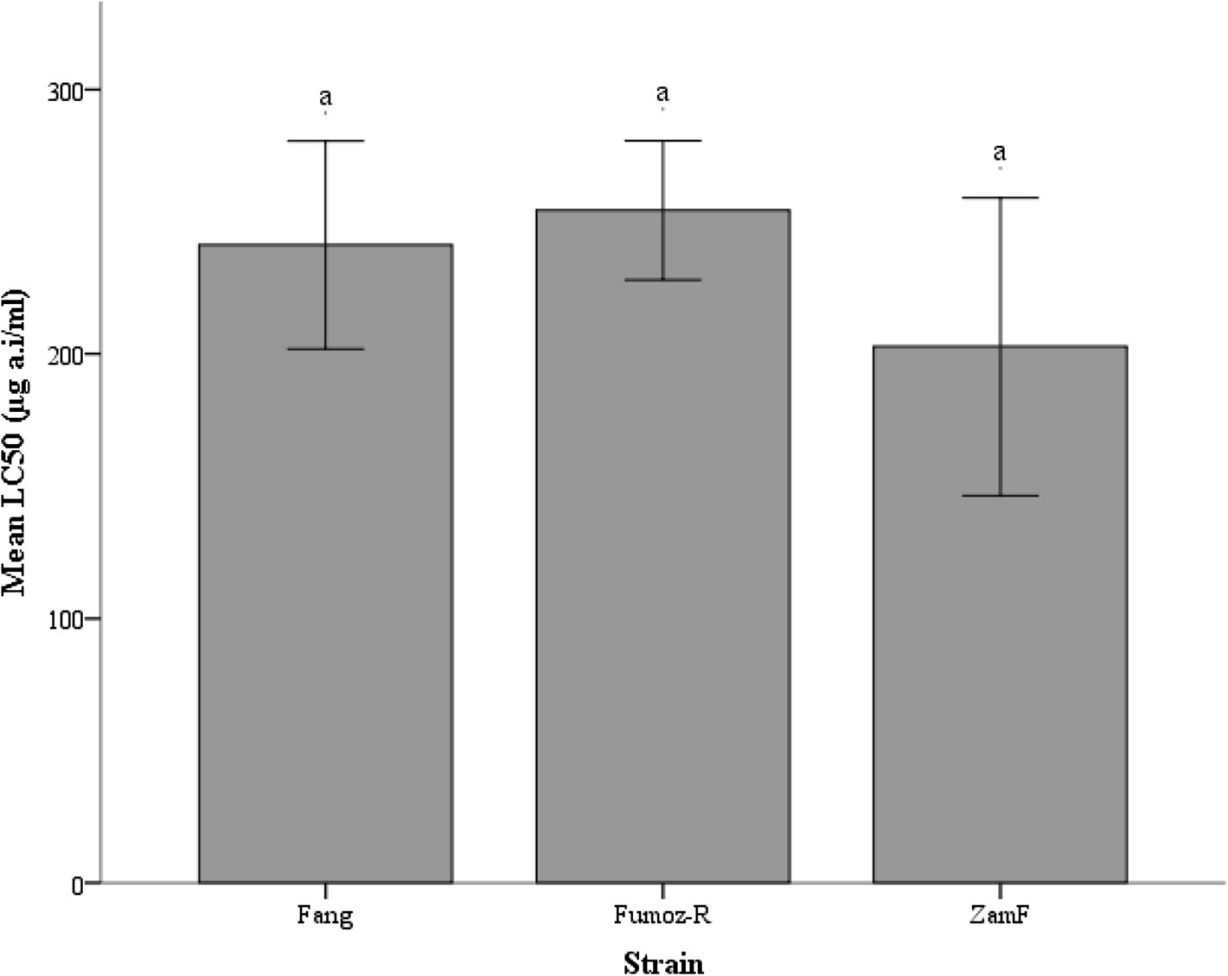
Mean lethal concentrations inducing 50 % mortality (LC50s) in *Anopheles funestus* strains 24 h post-exposure to C8910. Following C8910 exposure as per the CDC bottle bioassay protocol, LC50s were determined for each colony/wild-caught sample using regression lines of log-transformed mortality data. Analysis of variance (ANOVA) was used to compare LC50s between the three data sets

The mean lethal concentrations inducing 50 % mortalities (LC50s) in each sample are shown in Fig. 2. Although FUMOZ-R (254.39 ± 26.4 μg/ml a.i.) showed the highest mean LC50 followed by FANG (241.25 ± 39.4 μg/ml a.i.) and ZamF (202.80 ± 56.3 μg/ml a.i.), there was no significant variation in mean LC50 between samples (one-way ANOVA: F = 0.4, *p* = 0.69).

### Repellence tests

C8910 formulation 1 induced marked variation in mean landing responses between investigators (Table 1). For the FANG strain, significantly fewer landings were recorded on the control arm than the treatment arm of investigator 1. The reverse was true for investigator 2 for whom significantly fewer landings were recorded on the treatment arm than the control arm, while there was no significant difference in the mean numbers of landings on each arm of investigator 3. For the FUMOZ-R strain, no significant difference in the mean numbers of landings on each arm for any of the investigators was recorded.

**Table 1:** Mean number of mosquito landings per arm recorded during C8910 formulation 1 repellence assays

	Mean ± SE		*p*
	Control	Treatment	
FANG			
Investigator 1	10.4 ± 1.2	15.0 ± 1.1	0.01^a^
Investigator 2	17.4 ± 1.3	8.6 ± 1.4	0.03^a^
Investigator 3	9.0 ± 2.1	11.6 ± 1.5	0.44
All Investigators	12.3 ± 1.3	11.7 ± 1.0	0.80
FUMOZ-R			
Investigator 1	2.0 ± 0.9	3.2 ± 1.0	0.54
Investigator 2	4.6 ± 0.8	6.2 ± 1.5	0.47
Investigator 3	3.0 ± 0.3	1.0 ± 0.6	0.08
All Investigators	3.2 ± 0.5	3.5 ± 0.81	0.79

Standard choice-chamber bioassays were performed on three investigators using the laboratory-reared *Anopheles funestus* FANG (insecticide-susceptible) and FUMOZ-R (pyrethroid-resistant) strains. Each arm of an investigator acted as a control and treatment respectively. Results (p) of paired t-tests comparing control vs treatment arms are given

^a^indicates significant difference at 95 % confidence

C8910 formulation 2 also induced marked variation in mean landing responses between investigators (Table 2). For the FANG strain, significantly fewer landings were recorded on the control arm than the treatment arm of investigator 1. The reverse was true for investigator 2 for whom significantly fewer landings were recorded on the treatment arm than the control arm, while there was no significant difference in the mean numbers of landings on each arm of investigator 3. For the FUMOZ-R strain, significantly fewer landings were recorded on the treatment arm than the control arm of investigator 2 only. There were no significant differences in the mean numbers of landings on each arm of investigators 1 and 3. For the ZamF sample, there were no significant differences in the mean numbers of landings on each arm of both investigators using C8910 formulation 3 (Table 3).

**Table 2:** Mean number of mosquito landings per arm recorded during C8910 formulation 2 repellence assays

	Mean ± SE		*p*
	Control	Treatment	
FANG			
Investigator 1	2.4 ± 0.8	6.6 ± 0.9	0.02^a^
Investigator 2	8.6 ± 1.0	6.0 ± 0.6	0.03^a^
Investigator 3	8.6 ± 0.9	7.2 ± 1.2	0.42
All Investigators	6.5 ± 0.92	6.7 ± 0.62	0.85
FUMOZ-R			
Investigator 1	3.4 ± 1.0	4.0 ± 1.0	0.75
Investigator 2	6.4 ± 1.3	1.2 ± 0.5	0.03^a^
Investigator 3	2.4 ± 0.5	3.0 ± 0.5	0.21
All Investigators	4.1 ± 0.7	2.7 ± 0.5	0.22

Standard choice-chamber bioassays were performed on three investigators using the laboratory-reared *Anopheles funestus* FANG (insecticide-susceptible) and FUMOZ-R (pyrethroid-resistant) strains. Each arm of an investigator acted as a control and treatment respectively. Results (p) of paired t-tests comparing control vs treatment arms are given

^a^indicates significant difference at 95 % confidence

**Table 3:** Mean number of mosquito landings per arm recorded during C8910 formulation 3 repellence assays

	Mean ± SE		*p*
	Control	Treatment	
ZamF			
Investigator 1	3.6 ± 0.3	4.2 ± 0.4	0.21
Investigator 2	5.2 ± 0.7	6.6 ± 1.3	0.43
All investigators	4.4 ± 0.4	5.4 ± 0.8	0.24

Standard choice-chamber bioassays were performed on two investigators using the wild-caught *Anopheles funestus* ZamF samples. Each arm of an investigator acted as a control and treatment respectively. Results (p) of paired t-tests comparing control vs treatment arms are given

Repellence tests using DEET showed zero landings on the treatment arms of all investigators whereas multiple landings were recorded on the respective control arm of each investigator (Table 4).

**Table 4:** Mean number of mosquito landings per arm recorded during DEET repellence assays

	Mean ± SE		*p*
	Control	Treatment	
FANG			
Investigator 2	5.0 ± 0.6	0	0.01^a^
FUMOZ-R			
Investigator 1	4.0 ± 1.3	0	0.04^a^

Standard choice-chamber bioassays were performed on investigators using the laboratory-reared *Anopheles funestus* FANG (insecticide-susceptible) and FUMOZ-R (pyrethroid-resistant) strains. Each arm of an investigator acted as a control and treatment respectively. Results (p) of paired t-tests comparing control vs treatment arms are given

^a^indicates significant difference at 95 % confidence

## Discussion

In general, C8910 proved equally toxic to adult females of the pyrethroid resistant (FUMOZ-R) and insecticide susceptible (FANG) *An. funestus* laboratory strains, as well as to adult females of the wild-caught (ZamF) *An. funestus* sample. It should be noted, however, that the responses recorded for ZamF were more variable than those recorded for the laboratory strains. This likely reflects greater genetic heterogeneity in the wild-caught sample compared to the laboratory strains which are expected to show reduced genetic variation, and thereby reduced heterogeneity in response to insecticide intoxication, owing to laboratory colonisation and the effects of bottle-necking. The LD50 values recorded here for *An. funestus* are reasonably comparable to those recorded for other anophelines including *An. gambiae* (91.76 ug a.i./bottle), *An. dirus* (132.59 ug a.i./bottle), *An. farauti* (118.17 ug a.i./bottle), *An. freeborni* (119.87 ug a.i./bottle), *An. minimus* (55.44 ug a.i./bottle) and *An. stephensi* (112.58 ug a.i./bottle) [23], allowing for the fact that the exposure times differed substantially (30 min for *An. gambiae, An. dirus, An. farauti, An. freeborni, An. minimus* and *An. stephensi* vs two hours for *An. funestus*). Nevertheless, these data affirm the toxicity range of C8910 against *Anopheles* mosquitoes in general.

Based on CDC bottle bioassays similar to those used in the experiments described here, adult FUMOZ-R females show a 76.1 fold decrease in susceptibility to pyrethroids compared to FANG [27]. Pyrethroid resistance in FUMOZ-R is primarily mediated by the detoxifying capabilities of at least two monooxygenase P450s [8, 28–30] and the resistance phenotype is further enhanced by thickened cuticles [31]. The population from which the ZamF sample was derived also carries high levels of pyrethroid resistance [25]. Although the resistance mechanisms in this population have not been fully characterised as yet, preliminary data derived from synergist bioassays show that monooxygenases play a fundamentally important role. Collectively, the C8910 mortality data presented here suggest that monooxygenase-mediated pyrethroid resistance in *An. funestus* offers no protection against the toxic effects of C8910, and that this mixture of compounds therefore shows potential as an adulticide and as a “resistance breaker” for malaria vector control directed against *An. funestus*.

None of the C8910 formulations tested gave any conclusive indication of repellence against *An. funestus*. Although two of the formulations were scented (2 % original green leafy and 2 % modified green leafy fragrances), it is unlikely that the scent confounded the experiments because the unscented formulation gave similarly ambiguous results. It is possible that the 15 % formulations are too weak to induce repellence. These tests also suggest an investigator effect [32, 33] because only the data for investigator 2 indicated some repellence. However, compared to the data obtained using DEET, which showed complete repellence against *An. funestus* regardless of investigator, none of the tests using C8910 suggested a repellent effect. These data are surprising considering the demonstrations of repellence against other arthropods including *Aedes* and *Culex* mosquitoes [18–20, 22], and further investigations against *An. funestus* and other malaria vector species are required.

The fatty acids comprising C8910 have been approved by the U.S. FDA and are categorized as “Generally recognized as safe” [23]. They have low mammalian toxicity and are unlikely to pose significant environmental concerns. Further studies will be required to assess the residual activity of C8910 as an *An. funestus* adulticide. A promising mechanism for prolonging residual activity is micro-encapsulation, and various strategies are currently underway to produce formulations with extended activity [23]. The potential and efficacy of C8910 as a larvicide also requires investigation, and studies are currently underway to establish these for *An. funestus*.

## Conclusions

It is concluded that C8910 is equally effective as an adulticide against pyrethroid resistant and insecticide susceptible *An. funestus*. However, it did not show any consistent repellence against laboratory reared and wild-caught female samples of this species. Nevertheless, C8910 shows potential as an adulticide that can be used for malaria vector control, particularly in those instances where insecticide resistance management is required. However, future deployment as a public health intervention, most likely in conjunction with traditional insecticides in a mosaic or rotational strategy, will depend of further toxicity assessments followed by formulation, phased trial and commercialisation processes.

## References

[1] WHO (2014). World Malaria Report: 2014.

[2] Sinka ME, Bangs MJ, Manguin S, Coetzee M, Mbogo CM, Hemingway J, Patil AP, Temperley WH, Gething PW, Kabaria CW, Okara RM, Van Boeckel TP, Godfray HCJ, Harbach RE, Hay SI (2010). The dominant *Anopheles* vectors of human malaria in Africa, Europe and the Middle East: occurrence data, distribution maps and bionomic precis. Parasit Vectors.

[3] Gillies MT, Coetzee M (1987). A supplement to the Anophelinae of Africa south of the Sahara (Afrotropical Region).

[4] Harbach RE (2004). The classification of genus *Anopheles* (Diptera: Culicidae): a working hypothesis of phylogenetic relationships. Bull Entomol Res.

[5] Gillies MT, de Meillon B (1968). The Anophelinae of Africa south of the Sahara (Ethiopian zoogeographical region).

[6] Hemingway J, Ranson H (2000). Insecticide resistance in insect vectors of human disease. Annu Rev Entomol.

[7] Nkya TE, Akhouayri I, Kisinza W, David J-P (2013). Impact of environment on mosquito response to pyrethroid insecticides: facts, evidences and prospects. Insect Biochem Mol Biol.

[8] Coetzee M, Koekemoer LL (2013). Molecular systematics and insecticide resistance in the major African malaria vector *Anopheles funestus*. Annu Rev Entomol.

[9] Coetzee M, Knols BGJ, Louis C, Wageningen UR Frontis (2005). Malaria and Dengue Vector Biology and Control in Southern and Eastern Africa. Chapter 9. Bridging Laboratory and Field Research for Genetic Control of Disease Vectors.

[10] Coetzee M, Kruger P, Hunt RH, Durrheim DN, Urbach J, Hansford CF (2013). Malaria in South Africa: 110 years of learning to control the disease. S Afr Med J.

[11] Hargreaves K, Koekemoer LL, Brooke BD, Hunt RH, Mthembu J, Coetzee M (2000). *Anopheles funestus* resistant to pyrethroid insecticides in South Africa. Med Vet Entomol.

[12] WHO (2012). Global Plan for Insecticide Resistance Management in Malaria Vectors (GPIRM).

[13] Nauen R (2007). Insecticide resistance in disease vectors of public health importance. Pest Manag Sci.

[14] Zaim M, Guillet P (2002). Alternative insecticides: an urgent need. Trends Parasitol.

[15] Debboun M, Strickman D (2013). Insect repellents and associated personal protection for a reduction in human disease. Med Vet Entomol.

[16] Menger DJ, Van Loon JJA, Takken W (2014). Assessing the efficacy of candidate mosquito repellents against the background of an attractive source that mimics a human host. Med Vet Entomol.

[17] Stanczyk NM, Brookfield JFY, Field LM, Logan JG (2013). *Aedes aegypti* mosquitoes exhibit decreased repellency by DEET following previous exposure. PLoS One.

[18] Skinner WA, Tong H, Maibach H, Khan AA, Pearson T (1965). Repellency of skin-surface lipids of humans to mosquitoes. Science.

[19] Skinner WA, Tong HC, Maibach HI, Skidmore D (1970). Human skin-surface lipid fatty acids–mosquito repellents. Experientia.

[20] Hwang Y-S, Schultz GW, Axelrod H, Kramer WL, Mulla MS (1982). Ovipositional repellency of fatty acids and their derivatives against *Culex* and *Aedes* mosquitoes. Environ Entomol.

[21] Reifenrath WG. Pesticidal compositions for insects and arthropods. 2010. Patent no. WO 2010121142 A2. http://www.google.com/patents/WO2010121142A2?cl=en

[22] Mullens BA, Reifenrath WG, Butler SM (2009). Laboratory trials of fatty acids as repellents or antifeedants against houseflies, horn flies and stable flies (Diptera: Muscidae). Pest Manag Sci.

[23] Dunford JC, Wirtz RA, Reifenrath WG, Falconer A, Leite LN, Brogdon WG (2014). Determination of insecticidal effect (LCD50 and LCD90) of organic fatty acids mixture (C8910 + silicone) against malaria vectors. J Parasitol Vector Biol.

[24] Hunt RH, Brooke BD, Pillay C, Koekemoer LL, Coetzee M (2005). Laboratory selection for and characteristics of pyrethroid resistance in the malaria vector *Anopheles funestus*. Med Vet Entomol.

[25] Choi KS, Christian R, Nardini L, Wood OR, Agubuzo E, Muleba M, Munyati S, Makuwaza A, Koekemoer LL, Brooke BD, Hunt RH, Coetzee M (2014). Insecticide resistance and role in malaria transmission of *Anopheles funestus* populations from Zambia and Zimbabwe. Parasit Vectors.

[26] Brogdon W, Chan A (2010). Guidelines for Evaluating Insecticide Resistance in Vectors using the CDC Bottle Bioassay/ Methods in *Anopheles* Research. Second Edition. CDC Atlanta USA: CDC technical report.

[27] Wood OR, Spillings BL, Hunt RH, Koekemoer LL, Coetzee M, Brooke BD (2014). Sub-lethal pyrethroid exposure at the larval or adult life stage and selection for resistance in the major African malaria vector *Anopheles funestus* (Diptera: Culicidae). African Entomol.

[28] Brooke BD, Kloke G, Hunt RH, Koekemoer LL, Temu EA, Taylor ME, Small G, Hemingway J, Coetzee M (2001). Bioassay and biochemical analyses of insecticide resistance in southern African *Anopheles funestus* (Diptera: Culicidae). Bull Entomol Res.

[29] Amenya DA, Naguran R, Lo T-CM, Ranson H, Spillings BL, Wood OR, Brooke BD, Coetzee M, Koekemoer LL (2008). Over expression of a cytochrome P450 (CYP6P9) in a major African malaria vector, *Anopheles funestus*, resistant to pyrethroids. Insect Mol Biol.

[30] Irving H, Riveron JM, Ibrahim SS, Lobo NF, Wondji CS (2012). Positional cloning of rp2 QTL associates the P450 genes CYP6Z1, CYP6Z3 and CYP6M7 with pyrethroid resistance in the malaria vector *Anopheles funestus*. Heredity (Edinb).

[31] Wood O, Hanrahan S, Coetzee M, Koekemoer L, Brooke B (2010). Cuticle thickening associated with pyrethroid resistance in the major malaria vector *Anopheles funestus*. Parasit Vectors.

[32] Khan AA, Maibach HI, Strauss WG, Fenley WR (1965). Screening humans for degrees of attractiveness of mosquitoes. J Econ Entomol.

[33] Knols BG, de Jong R, Takken W (1995). Differential attractiveness of isolated humans to mosquitoes in Tanzania. Trans R Soc Trop Med Hyg.

